# Functional analysis of the novel *TBX5 *c.1333delC mutation resulting in an extended TBX5 protein

**DOI:** 10.1186/1471-2350-9-88

**Published:** 2008-10-01

**Authors:** Johann Böhm, Wolfram Heinritz, Alexander Craig, Mihailo Vujic, Britt-Marie Ekman-Joelsson, Jürgen Kohlhase, Ursula Froster

**Affiliations:** 1 Institut für Humangenetik und Anthropologie, Universität Freiburg, Freiburg, Germany; 2 Institut für Humangenetik, Medizinische Fakultät der Universität Leipzig, Leipzig, Germany; 3 Department of Clinical Genetics, Sahlgrenska University Hospital/East, Göteborg, Sweden; 4 Pediatric and Adolescent Medical Care, Skaraborg Hospital, Skovde, Sweden; 5 Praxis für Humangenetik, Freiburg, Germany

## Abstract

**Background:**

Autosomal dominant Holt-Oram syndrome (HOS) is caused by mutations in the *TBX5 *gene and is characterized by congenital heart and preaxial radial ray upper limb defects. Most of the *TBX5 *mutations found in patients with HOS cause premature truncation of the primary *TBX5 *transcript. *TBX5 *missense mutations alter the three-dimensional structure of the protein and result in failed nuclear localization or reduced binding to target DNA. In this study we present our functional analyses of the novel and unusual c.1333delC mutation found in a patient with classical HOS.

**Methods:**

The functional impact of this novel mutation was assessed by investigating the intracellular localization of the resulting TBX5 protein and its ability to activate the expression of its downstream target ANF.

**Results:**

The deletion of the cytosine is the first *TBX5 *frameshift mutation predicted to result in an elongated TBX5 protein with 74 miscoding amino acids and 62 supernumerary C-terminal amino acids. The c.1333delC mutation affects neither the nuclear localization, nor its colocalization with SALL4, but severely affects the activation of the ANF promoter.

**Conclusion:**

The mutation c.1333delC does not locate within functional domains, but impairs the activation of the downstream target. This suggests that misfolding of the protein prevents its biological function.

## Background

Holt-Oram syndrome (HOS) is an autosomal-dominantly inherited disorder caused by mutations in the gene coding for the T-box transcription factor TBX5, which is located on chromosome 12q24 [1,2]. Clinical features are highly penetrant congenital skeletal malformations of the upper limbs associated with variable cardiac anomalies including ASD, VSD, AVSD, PDA, tetralogy of Fallot, heart conduction failure and pulmonary vein anomalies [3]. To date, more than 60 different germline *TBX5 *gene mutations have been described (*TBX5 *gene mutation database; ) [4]. A vast majority of these mutations (nonsense, frameshift and splice site mutations) result in preterminal stop codons leading to truncated nonfunctional proteins. Also larger deletions spanning one or more exons have been reported [5,6]. Nonsyndromic germline mutations are not known [7], but somatic mutations (exclusively missense mutations) were found in non-HOS related malformed hearts [8]. The detection rate of mutations in the *TBX5 *gene in patients with familial or sporadic HOS is 74% following application of stringent clinical criteria [9].

TBX5 contains a T-box motif involved in DNA-binding and protein-protein interactions, and operates as a transcriptional activator [10,11]. Mutations within the T-box affect DNA-binding and interaction with NKX2-5, resulting in reduced activation of downstream targets in the cardiac conduction system development [12,13]. In the mouse, Tbx5 controls *Fgf10 *expression in a synergistic manner together with Sall4 in the forelimbs via direct effects on the *Fgf10 *promoter [14]. This cooperative action of Tbx5 and Sall4 can be counteracted by Tbx2 and Tbx3. Furthermore, TBX5 was shown to interact with GATA4. Specific missense mutations within *TBX5 *abrogate this interaction and cause cardiac septal defects [15]. Transactivation of TBX5 is promoted by TAZ, a protein complexing TBX5 and interacting with the histone acetyltransferase p300 [16]. During embryogenesis TBX5 localizes to cellular nuclei. The protein transport is mediated by two distinct, evolutionary conserved nuclear localization signals (NLS) acting cooperatively [17]. Missense mutations within these conserved sequences impair nuclear localization and cause "functional" haploinsufficiency due to decreased protein concentration at the location of its biological function [13].

Here we report on the results of functional analyses of a *de novo *c.1333delC mutation identified in a patient with sporadic HOS. This mutation does not impair nuclear localization or co-localization with SALL4, but abrogates activation of the ANF promoter.

## Methods

### Case report

**Figure 1:**
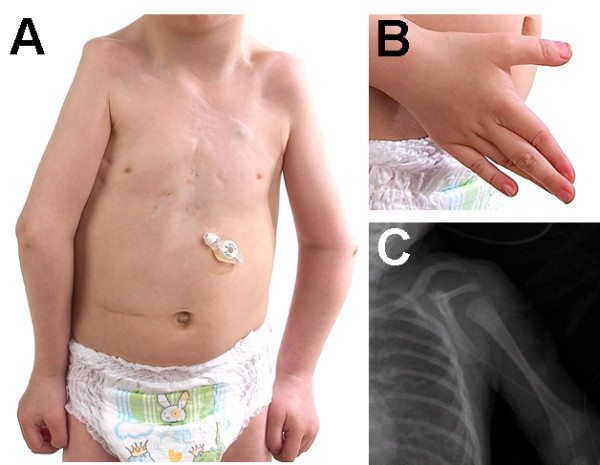
**The patient**. The patient presents bilateral hypoplastic clavicles and radii (A and C) and bilateral triphalangeal thumbs (B). Bradycardia due to atrioventricular block necessitates a pacemaker.

The now 4-year old male patient (Fig. 1) presented with typical manifestations of Holt-Oram syndrome: bilateral hypoplastic clavicles and radii (Fig. 1A, 1C), bilateral triphalangeal thumbs (Fig. 1B), total ASD and muscular VSD, bradycardia due to atrioventricular block requiring pacemaker, hypoplastic lung and pulmonary veins on the right side. He also had micrognathia and a long philtrum. Mental development is normal up to now.

### Genetic analysis

After obtaining informed consent, genomic DNA was extracted from peripheral blood lymphocytes of the index patient and his unaffected parents according to standard protocols. Mutation analysis of the coding sequence of the *TBX5 *gene including exon-intron boundaries was performed by direct DNA sequencing as described [18].

### Plasmids

*TBX5 *expression constructs were generated by PCR amplification using the commercially available human full-length cDNA clone (IRATp970D0542D, RZPD, Berlin, Germany). The sequence of the constructs was verified by automated sequencing (ABI Prism^® ^3100 Genetic Analyzer, Applied Biosystems, Darmstadt, Germany). PCR fragments were subcloned into the pGEM^®^-T-Easy vector (Promega, Madison, WI, USA), digested with *EcoR*I and *BamH*I and subsequently cloned into the pEGFP-N3/pFLAG-N3 vectors (BD Biosciences, Mountain View, CA, USA). The ANF-luciferase vector was a kind gift from Benoit Bruneau (Hospital for Sick Children, Toronto, Canada).

### Generation of the c.1333delC mutation

The *TBX5 *construct carrying the c.1333delC mutation was generated with the QuikChange™ Site-directed Mutagenesis kit (Stratagene, La Jolla, CA, USA). Successful incorporation of the mutations was confirmed by automated sequencing.

### Cell culture

COS-7 cells were grown in Minimal essential Medium (MEM), 10% Fetal Calf Serum, 100 μg/ml penicillin, 100 μg/ml streptomycin, 1× MEM nonessential amino acids (all purchased from Invitrogen, Carlsbad, CA, USA) and maintained in a humidified environment with 5% carbon dioxide.

### Intracellular localization

Co-transfected COS-7 cells were directly seeded on glass coverslips in 24-well plates (1 × 10^4 ^cells per plate), washed in PBS after 24 h, fixed with methanol, washed in PBS and blocked for 30 min in 0.5% BSA in IF buffer (10 mM Tris pH 7.5, 300 mM NaCl, 0.05% (v/v) Tween 20). For the detection of intracellular SALL4 localization, coverslips were incubated with a monoclonal anti-FLAG^® ^M2 antibody (Stratagene, La Jolla, CA, USA) for 1 h, washed twice with IF buffer and incubated with a fluorescence-labeled anti-mouse IgG (Fab specific)-TRITC antibody for 30 min. Cells were washed three times with IF buffer and mounted on slides with aquamount (BDH Laboratories, Poole, UK). Where indicated, cell nuclei were stained with DAPI blue. The TBX5 distribution pattern could be visualized due to fluorescence of the GFP fusion protein. Cells were analyzed by confocal laser scanning microscopy (CLSM).

### Reporter gene assays

The wild type and mutated *TBX5 *cDNAs were cloned into the mammalian expression vector pFLAG-N3. The reporter construct contained the luciferase gene under the control of the atrial natriuretic factor (ANF) promoter. Each transfection was performed at least in triplicate in 24-well plates and all assays were done with two different DNA preparations of each construct. Transfections of adherent COS-7 cells were carried out at 60–80% confluency using 1 μg Lipofectamine™2000 reagent (Invitrogen, Carlsbad, CA, USA) per 500 ng transfected DNA. In each transfection an equimolar DNA concentration was used. Control vector pRL-SV40 (Promega, Madison, WI, USA), expressing the *Renilla reniformis *luciferase, was co-transfected for relativating transfection efficiency at a ratio of 1:100 to the total amount of transfected DNA. In order to analyze the transcriptional regulation capability of the *TBX5 *constructs, 300 ng *ANF*-luciferase plasmid and 300 ng TBX5 expression vector were used for co-transfections. DNAs of empty expression vector plasmids were used as negative control. Cell extracts were harvested 24 hours after transfection and dissolved in 125 μl Passive Lysis Buffer (Promega) per well. 20 μl were used to measure luciferase activity according to the Dual Luciferase Assay System Protocol (Promega) using a Lumat LB96P luminometer (Berthold, Bad Wildbad, Germany).

## Results

### Mutation analysis

We identified a novel heterozygous mutation in exon 9 of *TBX5 *(c.1333delC, p.H445*fs*X136) in the index patient, but not in his parents. The *de novo *deletion of the cytosine residue at cDNA position 1333 (codon 445) is predicted to result in a translational frameshift creating not a truncated but an elongated TBX5 protein of 580 amino acids due to a downstream shift of the termination codon to position 581 (normal codon position 519). Thus, the mutant TBX5 protein would contain 74 miscoding amino acids (positions 445 to 518) and 62 supernumerary C-terminal amino acids [see Additional file 1]. These findings suggested a *de novo *mutational event, which most likely caused Holt-Oram syndrome in our patient. To provide evidence for the pathogenic effect of this special mutation, we carried out further functional investigations.

### c.1333delC does not influence intracellular localization

The entire coding sequence of human wild type and mutated *TBX5 *was cloned into the GFP expression plasmid pEGFP-N3, whereas the coding sequence of *SALL4 *was inserted into the FLAG expression plasmid pFLAG-N3, resulting in TBX5wt-GFP, TBX5mut-GFP and SALL4-FLAG fusion proteins. TBX5 was shown to display a nuclear localization during embryogenesis, thereby having two distinct nuclear localization signals working cooperatively [17,19]. The murine homologue of *SALL4 *is a nuclear protein localized to heterochromatin and operating as a transcriptional repressor [20]. In the mouse, Sall4 and Tbx5 colocalize and interact physically [14].

**Figure 2:**
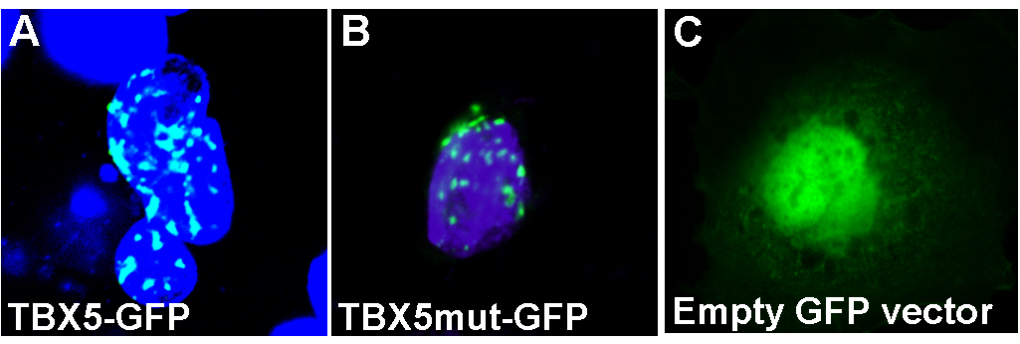
**Intracellular localization of wild type and mutant TBX5**. (A) COS-7 cells were transfected with *TBX5 *fused to the sequence encoding the green fluorescent protein (GFP). Wild type TBX5 appeared in dot-like structures in the nuclei (blue, stained with DAPI) of the analyzed cells. (B) The mutant TBX5 displays a distinct and homogenous arrangement in the nucleus, the distribution pattern does not differ compared to the wild type TBX5. (C) The empty pEGFP-N3-vector was transfected as a negative control and resulted in a diffuse nuclear staining.

**Figure 3:**
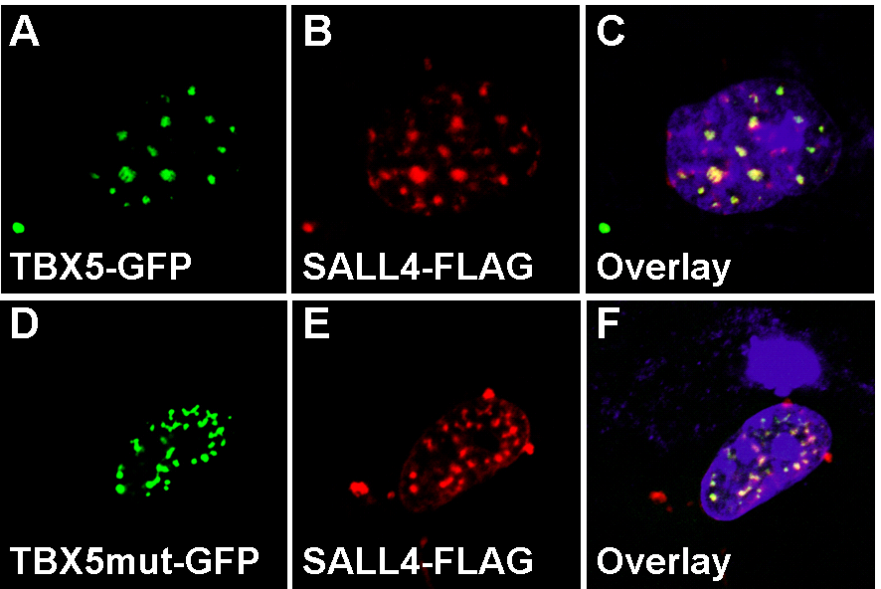
**Co-localization of wild type and mutant TBX5 with SALL4**. The subcellular distribution of the proteins was analyzed in COS-7 cells transfected with either wild type or mutant *TBX5*-GFP constructs and *SALL4*-FLAG. SALL4 was detected using mouse α-FLAG antiserum and a secondary goat anti-mouse TRITC-labelled antibody. SALL4 and TBX5 (A, B, C) form overlapping distinct structures in the nucleus. The SALL4 signal was also shown to interfere with subcellular distribution of the mutated TBX5 in dot-like structures in the nucleus (D, E, F). The nuclei, stained with DAPI, appear in blue.

In order to analyze the mutated TBX5 protein for possible impaired nuclear localization we transfected COS-7 cells with TBX5-GFP wild type or mutant constructs and analyzed the intracellular distribution of the proteins by use of confocal laser scanning microscopy (CLSM, Fig. 2). The distribution of TBX5 wild type (Fig. 2A) and mutant proteins (Fig. 2B) both appeared as dot-like structures in the nucleus. Transfection of the empty GFP vector was used as negative control, and resulted in a diffuse nuclear staining (Fig. 2C). To assess whether the intranuclear distribution of mutated TBX5 differs from the wild type protein, we co-transfected COS-7 cells with *SALL4 *and either wild type or mutated *TBX5 *constructs. SALL4 was detected by use of mouse anti-FLAG antibody and goat anti-mouse IgG-TRITC. An overlay of the SALL4 and TBX5 wild type (Fig. 3A, 3B, 3C) and mutant signals (Fig. 3D, 3E, 3F) demonstrated co-localization of both proteins, respectively. Thus, the mutation c.1333delC does not affect the transport of the protein to the nucleus nor its intranuclear distribution.

### c.1333delC mutation impairs activation of the ANF promoter

**Figure 4:**
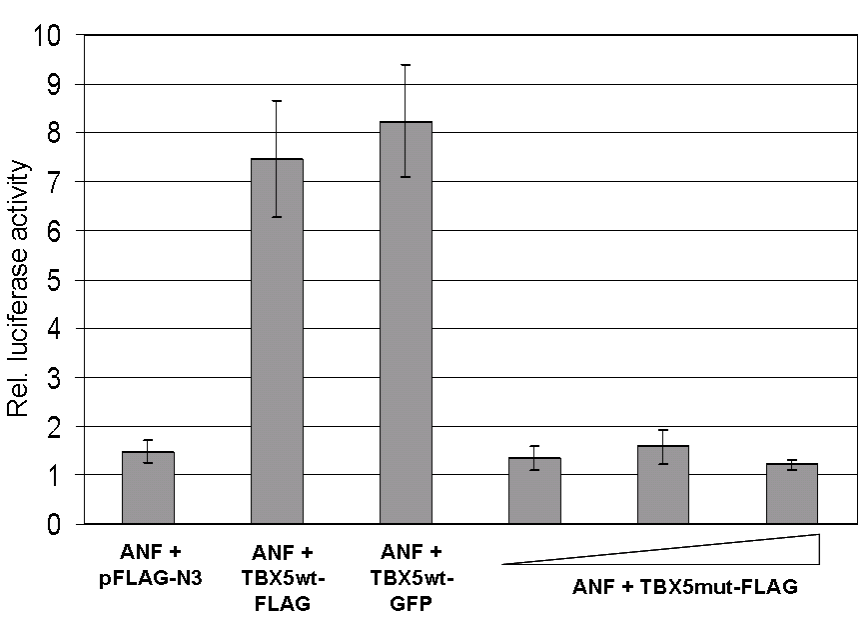
**Functional effect of the *TBX5 *c.1333delC mutation on transcriptional regulation**. COS-7 cells were transiently co-transfected either with mutant or wild type *TBX5 *expression constructs and a reporter encoding the luciferase gene under the control of the *ANF *promoter. Values were calculated by normalizing against *Renilla reniformis *luciferase activity and compared to the empty FLAG vector (column 1). Wild type TBX5-FLAG and TBX5-GFP effectively induced expression of the reporter gene (columns 2 and 3), whereas cells transfected with rising amounts of the mutant *TBX5 *construct demonstrated entire abrogation of reporter gene activation compared to wild type *TBX5 *(column 4, 5, 6).

An important functional domain of TBX5 is the T-box motif involved in DNA-binding and protein-protein interactions [10,11]. The majority of the described *TBX5 *mutations result in preterminal stop codons. The functional effect of the c.1333delC mutation – leading to an elongated protein – on transcriptional regulation was assessed using a luciferase assay. COS-7 cells were transiently co-transfected with mutant/wild type *TBX5 *expression constructs (pFLAG-N3) and an *ANF*-luciferase construct as reporter. The upstream region of the *ANF *(atrial natriuretic factor gene) promoter contains a TBX5 binding site [21]. The luciferase activity was normalized in reference to the *Renilla reniformis *luciferase activity, used as an internal control. The constructs were analyzed by immunoblot for appropriate expression (data not shown). Reporter gene activation significantly increased in cells transfected with wild type *TBX5 *compared to cells transfected with the empty expression vector. Transfection of the *TBX5*-GFP construct lead to the same result, indicating that neither the FLAG-tag nor the GFP-tag (as used in the localization studies) have a detrimental effect on the protein function. The luciferase activity of the mutated *TBX5*-FLAG construct was strongly reduced to a value comparable to cells transfected with the empty pFLAG-N3 vector. An increased amount of transfected *TBX5*mut plasmid (300 ng, 450 ng, 600 ng) did not induce luciferase activity, ruling out a dosage effect (Fig. 4). Under the experimental conditions of cell culture mediated luciferase assays, the mutation c.1333delC abrogates activation of the *ANF *promoter by TBX5.

## Discussion

Autosomal dominant Holt-Oram syndrome, linked to mutations in the T-box transcription factor gene *TBX5*, is characterized by bilateral forelimb malformations and congenital heart defects. Also defects of the pulmonary veins are common. Mutations found in *TBX5 *include missense, nonsense, frameshift and splice site mutations as well as large deletions (*TBX5 *gene mutation database; ) [4,6,22]. Mutations in the N-terminal region of the T-box affect binding of TBX5 to the major groove of DNA, while mutations in the C-terminal region of the T-box affect binding to the minor grove. There is no obvious genotype-phenotype correlation. The severity of limb and heart varies independently of type and location of the mutation [23]. Diverse known *TBX5 *missense mutations (p.Q49K, p.I54T, p.G80R, p.G169R, p.R237Q, p.R237W, and p.S252I) reduce synergistic transcriptional activity of the downstream target *ANF*, but only the mutations p.G80R, p.R237Q, and p.R237W were shown to affect DNA-binding [13]. In addition, an impaired nuclear localization of the altered proteins was demonstrated. Similar results were obtained by functional analysis of the in-frame mutation 381-408del (c.376_402del, p.K126_R134del) [13]. To assess a putative functional effect of the *de novo *c.1333delC (p.H445*fs*X136) mutation on transcriptional activation we co-transfected COS-7 cells with mutant/wild type *TBX5 *expression constructs and an *ANF*-luciferase construct. Under the experimental conditions of the luciferase assay, the reporter gene activation through the mutated TBX5 protein was abrogated and non functional as compared to the wild type protein.

The human *TBX5 *gene consists of 8 coding exons and one 5' non-coding exon dispersed over 53 kilobases (kb) on chromosome 12q24. Proteins of the T-box family comprise two distinct structural and functional domains: the DNA-binding domain, referred as T-box, and a transcriptional activator or repressor domain [24]. Residues of the T-box are highly conserved throughout members of this family and across species. Binding-site selection experiments showed an affinity of T-box proteins for the consensus sequence TCACACCT [25]. The transcriptional regulation activity was shown to require sequence motifs within the C-terminal part of the protein [25]. For the development of specific organs like heart and upper limbs, TBX5 is an essential transcription factor. The import to the nucleus is thereby promoted by two distinct nuclear localization signals (NLS) working in concert. The two minimal sequences required for nuclear import were found to reside within (aa 79–90) and carboxyl to the T-box (325–340). The first amino acid affected by the mutation c.1333delC (His445) is carboxyterminal of both regions required for nuclear localization. This mutation resides within the last exon, we therefore assume, that the *TBX5 *mRNA bearing the single nucleotide deletion is not degraded by nonsense mediated decay. This mechanism was shown to occur only in case that the preterminal stop codon is located at least 50–55 nucleotides before the last exon/exon boundary [26]. Thus, messenger RNAs with nonsense mutations or small deletions/insertions in the last exon are generally translated into a protein. As the patient's deletion was detected in the last exon of *TBX5*, the mRNA is predicted to result in a elongated protein consisting of 580 instead of 518 amino acids. It is conceivable that an altered protein configuration or steric hindrance may silence the activity of the NLS motifs. We therefore wanted to analyze the intracellular distribution of the mutated TBX5 protein. *TBX5 *is mainly expressed during embryonic morphogenesis and is limited to esophagus, heart, liver, lung, prostate and trachea in the adult organism. Blood samples from the patient were taken for DNA sequence analysis, but could not be used for investigations on the TBX5 protein as it is not detectable in lymphocytes. Consequently we did not have the possibility to analyze the presence and the intracellular localization of the endogenous elongated protein. Other possible TBX5 sources were not available. All experiments were therefore carried out by transfecting wild type and mutated fusion constructs into an appropriate cell line.

Both wild type as well as mutated TBX5 proteins were found to localize in dot-like structures within the nucleus, and both co-localize with SALL4. The murine homologue Sall4 was previously demonstrated to localize to heterochromatin and to operate as a transcriptional repressor [20].

## Conclusion

Interestingly, four other truncating mutations have been detected in exon 9 of the *TBX5 *gene (c.1024delT, c.1084C>T, c.1159dupA, c.1366C>T) so far to our knowledge. All result in shortening of the TBX5 protein. However, it is unclear how those lead to TBX5 haploinsufficiency, since the mutations will most likely not lead to nonsense-mediated mRNA decay, because they reside within the most 3' positioned exon. The unique mutation c.1333delC differs from the other mutations in exon 9 as it leads to an elongated protein. Since the mutation neither affects the intranuclear distribution of TBX5 nor its colocalization with SALL4, but abrogates activation of the ANF promoter, it is tempting to speculate that the supernumerary amino acids modulate the configuration of the protein by masking the transcriptional activator domain.

## Competing interests

The authors declare that they have no competing interests.

## Authors' contributions

JB conceived of the study, carried out the functional molecular genetic studies and drafted the manuscript. WH and UF accomplished the sequence analysis and wrote the clinical section of the manuscript. AC participated in the functional molecular genetic studies. MV and BMEJ collected the clinical data. JK coordinated the project and participated in the manuscript preparation.

## Consent

Written consent for publication was obtained from the patient's parents.

## Pre-publication history

The pre-publication history for this paper can be accessed here:



## Supplementary Material

Additional file 1 **The c.1333delC mutation results in an elongated protein**. The heterozygous c.1333delC mutation (yellow) in exon 9 of *TBX5 *results in a translational frameshift creating an elongated TBX5 protein due to a downstream shift of the termination codon. The mutant TBX5 protein contains 74 miscoding amino acids and 62 supernumerary C-terminal amino acids (black letters). Click here for file
