# Supplementary material for: Functional analysis of the novel TBX5 c.1333delC mutation resulting in an extended TBX5 protein
Source: BMC Med Genet. 2008 Oct 1;9:88. doi: 10.1186/1471-2350-9-88 (PMC2567295; doi:10.1186/1471-2350-9-88)
Supplement: Additional file 1 — The c.1333delC mutation results in an elongated protein. The heterozygous c.1333delC mutation (yellow) in exon 9 of TBX5 results in a translational frameshift creating an elongated TBX5 protein due to a downstream shift of the termination codon. The mutant TBX5 protein contains 74 miscoding amino acids and 62 supernumerary C-terminal amino acids (black letters). [file 1471-2350-9-88-S1.pdf]

```
atggccgacgcagacgagggctttggcctggcgcacacgcctctggagcctgacgcaaaagacctgccctgcgattcgaaacccgagagcgcg
·M··A··D··A··D··E··G··F··G··L··A··H··T··P··L··E··P··D··A··K··D··L··P··C··D··S··K··P··E··S··A·

ctcggggcccccagcaagtccccgtcgtccccgcaggccgccttcacccagcagggcatggagggaatcaaagtgtttctccatgaaagagaa
·L··G··A··P··S··K··S··P··S··S··P··Q··A··A··F··T··Q··Q··G··M··E··G··I··K··V··F··L··H··E··R··E·

ctgtggctaaaattccacgaagtgggcacggaaatgatcataaccaaggctggaaggcggatgtttcccagttacaaagtgaaggtgacgggc
·L··W··L··K··F··H··E··V··G··T··E··M··I··I··T··K··A··G··R··R··M··F··P··S··Y··K··V··K··V··T··G·

cttaatcccaaaacgaagtacattcttctcatggacattgtacctgccgacgatcacagatacaaattcgcagataataaatggtctgtgacg
·L··N··P··K··T··K··Y··I··L··L··M··D··I··V··P··A··D··D··H··R··Y··K··F··A··D··N··K··W··S··V··T·

ggcaaagctgagcccgccatgcctggccgcctgtacgtgcacccagactcccccgccaccggggcgcattggatgaggcagctcgtctccttc
·G··K··A··E··P··A··M··P··G··R··L··Y··V··H··P··D··S··P··A··T··G··A··H··W··M··R··Q··L··V··S··F·

cagaaactcaagctcaccaacaaccacctggacccatttgggcatattattctaaattccatgcacaaataccagcctagattacacatcgtg
·Q··K··L··K··L··T··N··N··H··L··D··P··F··G··H··I··I··L··N··S··M··H··K··Y··Q··P··R··L··H··I··V·

aaagcggatgaaaataatggatttggctcaaaaaatacagcgttctgcactcacgtctttcctgagactgcgtttatagcagtgacttcctac
·K··A··D··E··N··N··G··F··G··S··K··N··T··A··F··C··T··H··V··F··P··E··T··A··F··I··A··V··T··S··Y·

cagaaccacaagatcacgcaattaaagattgagaataatccctttgccaaaggatttcggggcagtgatgacatggagctgcacagaatgtca
·Q··N··H··K··I··T··Q··L··K··I··E··N··N··P··F··A··K··G··F··R··G··S··D··D··M··E··L··H··R··M··S·

agaatgcaaagtaaagaatatcccgtggtccccaggagcaccgtgaggcaaaaagtggcctccaaccacagtcctttcagcagcgagtctcga
·R··M··Q··S··K··E··Y··P··V··V··P··R··S··T··V··R··Q··K··V··A··S··N··H··S··P··F··S··S··E··S··R·

gctctctccacctcatccaatttggggtcccaataccagtgtgagaatggtgtttccggcccctcccaggacctcctgcctccacccaaccca
·A··L··S··T··S··S··N··L··G··S··Q··Y··Q··C··E··N··G··V··S··G··P··S··Q··D··L··L··P··P··P··N··P·

tacccactgccccaggagcatagccaaatttaccattgtaccaagaggaaagaggaagaatgttccaccacagaccatccctataagaagccc
·Y··P··L··P··Q··E··H··S··Q··I··Y··H··C··T··K··R··K··E··E··E··C··S··T··T··D··H··P··Y··K··K··P·

tacatggagacatcacccagtgaagaagattccttctaccgctctagctatccacagcagcagggcctgggtgcctcctacaggacagagtcg
·Y··M··E··T··S··P··S··E··E··D··S··F··Y··R··S··S··Y··P··Q··Q··Q··G··L··G··A··S··Y··R··T··E··S·

gcacagcggcaagcttgcatgtatgccagctctgcgcccccagcgagcctgtgcccagcctagaggacatcagctgcaacacgtggccaagc
·A··Q··R··Q··A··C··M··Y··A··S··S··A··P··P··S··E··P··V··P··S··L··E··D··I··S··C··N··T··W··P··S·

atgccttcctacagcagctgcaccgtcaccaccgtgcagcccatggacaggctaccctaccagcacttctccgctcacttcacctcggggccc
·M··P··S··Y··S··S··C··T··V··T··T··V··Q··P··M··D··R··L··P··Y··Q··H··F··S··A··H··F··T··S··G··P·

ctggtccctcggctggctggcatggccaaccatggctccccacagctgggagagggaatgttccagcaccagacctccgtggcccaccagcct
·L··V··P··R··L··A··G··M··A··N··H··G··S··P··Q··L··G··E··G··M··F··Q··H··Q··T··S··V··A··H··Q··P·
                                 M··A··P··H··S··W··E··R··E··C··S··S··T··R··P··P··W··P··T··S··L·

gtggtcaggcagtgtgggcctcagactggcctgcagtcccctggcacccttcagccccctgagttcctctactctcatggcgtgccaaggact
·V··V··R··Q··C··G··P··Q··T··G··L··Q··S··P··G··T··L··Q··P··P··E··F··L··Y··S··H··G··V··P··R··T·
 ·W··S··G··S··V··G··L··R··L··A··C··S··P··L··A··P··F··S··P··L··S··S··S··T··L··M··A··C··Q··G··L·

ctatcccctcatcagtaccactctgtgcacggagttggcatggtgccagagtggagcgacaatagctaaagtgaggcctgcttcacaacagac
·L··S··P··H··Q··Y··H··S··V··H··G··V··G··M··V··P··E··W··S··D··N··S··*·
 ·Y··P··L··I··S··T··T··L··C··T··E··L··A··W··C··Q··S··G··A··T··I··A··K··V··R··P··A··S··Q··Q··T·

atttcctagagaaagagagagagagaggagaaagagagagaaggagagagacagtagccaagagaaccccacggacaagatttttcatttcac
 ·F··P··R··E··R··E··R··E··R··R··K··R··E··K··E··R··D··S··S··Q··E··N··P··T··D··K··I··F··H··F··T·

ccaatgttcacatctgcactcaaggtcgctggatgctgatctaatcagtagcttgaaaccacaattttaa
 ·Q··C··S··H··L··H··S··R··S··L··D··A··D··L··I··S··S··L··K··P··Q··F··*·
```
